# Supplementary material for: Restaurant staff’s knowledge, practices, and attitudes pertaining to food allergy in Qassim region, Saudi Arabia: A cross-sectional analysis
Source: PLoS One. 2024 Oct 23;19(10):e0311650. doi: 10.1371/journal.pone.0311650 (PMC11498656; doi:10.1371/journal.pone.0311650)
Supplement: Attachment — Submitted filename: renamed_10695.docx [file pone.0311650.s001.docx]

Thank you for your thorough review of our submission. We appreciate your feedback and have addressed the issues raised regarding the consent procedures. In the revised Ethics Method section and manuscript, we have clarified the following:

The consent procedure has been done through two steps. The first step is by contacting the restaurant owner via the phone (The only contact method available) and obtaining their consent to visit the restaurant and interview its employees. Once we obtained the first consent, we move to obtain the consent from the restaurant employees verbally. The reason for opting the verbal consent is due to the lack of reading abilities of the interviewed participants due to different languages. However, verbal communication with this population is effective and easier. Due to the large sample size and the type of data collected which does not involve any sensitive and identifiable information, no documentation except the witness of two research assistants was made.

We believe these revisions comprehensively address your concerns. Please let us know if further clarification or information is required.

Thank you for your time and consideration.

Best regards,

Norah Aljuaylan
