# Supplementary material for: Restaurant staff’s knowledge, practices, and attitudes pertaining to food allergy in Qassim region, Saudi Arabia: A cross-sectional analysis
Source: PLoS One. 2024 Oct 23;19(10):e0311650. doi: 10.1371/journal.pone.0311650 (PMC11498656; doi:10.1371/journal.pone.0311650)
Supplement: Attachment — Submitted filename: Review PLOS.docx [file pone.0311650.s002.docx]

Line 34-35: Change word “omitted”with another word such as “did not list” or “did not post” if its about food allergy information not being listed for the guests.

Line 36: change “varies” to “varied” as the research is complete, so it should be written in past tense.

Line 38: say “food allergy measures” you do not have to say food safety.

Line 59: add sesame to the list of allergens. It is now the 9^th^ allergen on the list of top allergens.

Line 119: remove word “are”. Replace with “were”

Line 151: Remove word “we”.

Table 1: I suggest you divide the demographic data in more detail. Example: Non-Asian restaurants can be of so many cuisines.

Foodservice should be one word.

Discussion section is very short. It should have more discussion. You have enough data to discuss them in detail. Include more references to discuss results.
