# Supplementary material for: Restaurant staff’s knowledge, practices, and attitudes pertaining to food allergy in Qassim region, Saudi Arabia: A cross-sectional analysis
Source: PLoS One. 2024 Oct 23;19(10):e0311650. doi: 10.1371/journal.pone.0311650 (PMC11498656; doi:10.1371/journal.pone.0311650)
Supplement: Attachment — Submitted filename: Response to Reviewers.rtf [file pone.0311650.s003.rtf]

Based on the reviewers comment #5 " The authors need to add more references and comprehensively discuss the results, and clearly identify limitations, future research.", the results and discussion has been revised. We have answered the question asked by reviwer2 below:
The question:
The manuscript presented in an intelligible fashion and written in standard Language. I have the following queries or suggestions: Firstly, the authors should identify what is meant by " scored in the top 50%". On what bases you choose 50%?

Our answer:
For the knowledge score, we summed the number of correct answers (out of 19) and used each group's median score to dichotomize the participants as having more or less knowledge.
For the attitude score, we assigned point values to each response as follows: strongly disagree = 1, disagree = 2, unsure = 3, agree = 4, and strongly agree = 5. We then averaged each participant's response to the five attitude questions. We used each group's median score to divide participants into those having relatively positive or less positive attitudes. This has been added to the methodology section.

Line (394-89) and Table 1 We changed Food service to Foodservice.
Line 35: We changed (omitted) to (did not post)
Line 36: We changed (varies) to (varied)
Line 38: We changed (food safety) to (food allergy measures)
Line 59: We add sesame to the list of allergens.
Line 151: Remove the word (we) 
Additionally, Table 6 has been added as it was observed that overall knowledge was not measured. This is very important to determine the need for additional training courses.


About this point (Table 1: I suggest you divide the demographic data in more detail. Example: Non-Asian restaurants can be of so many cuisines) A good suggestion, but unfortunately, we cannot make the adjustment now as the data was collected based on the original classification.

Response to the Ethical Considerations Regarding Obtaining the Consent:
Thank you for your inquiry regarding obtaining the consent. A waiver of documentation of informed consent is requested in accordance with 45 CFR 46.117(c). The proposed research meets the first criterion for the waiver, as the probability and magnitude of harm or discomfort in participation are not greater in and of themselves than those ordinarily encountered in daily life. Additionally, as the
research involves no procedures for which written consent is normally required outside the research context, the study also meets the second criterion for waiver. Before conducting the study, we obtained verbal informed consent from the restaurant managers. 
The member read for the manager a short introduction describing the purpose of the
study and how the data will be used. Then, the interviewer asked the manager if he or she agrees to participate in the study. The interview proceeded for those who agreed to participate. The interviewer read a brief recruiting and informed consent script to the worker/server who was identified as a potential participant by the manager. We requested a waiver of written informed consent; thus, neither managers nor workers/servers read the informed consent scripts.
Kind regards,
Norah Aljuaylan
