# Supplementary material for: Restaurant staff’s knowledge, practices, and attitudes pertaining to food allergy in Qassim region, Saudi Arabia: A cross-sectional analysis
Source: PLoS One. 2024 Oct 23;19(10):e0311650. doi: 10.1371/journal.pone.0311650 (PMC11498656; doi:10.1371/journal.pone.0311650)
Supplement: Attachment — Submitted filename: report.docx [file pone.0311650.s004.docx]

**Reviewer report for PLOS one Journal**

Title: Restaurant staff’s knowledge, practices, and attitudes pertaining to food allergy in

Qassim region, Saudi Arabia: A cross-sectional analysis

1. **Abstract:**
   1.1. In abstract, method section, the statement: (Among the 178 restaurants included in the analysis, 97% of the managers and servers were men, and most had not completed food allergy courses. Notably, 86% of the participants omitted any form of food allergy information from their operations.) could be removed and put another main finding.
   1.2. The abstract section should include the main findings in concise manner.
   1.3. The result section in abstract is deficient, it is not supported by enough findings.
2. **Introduction:** suitable and expressive. The objectives are clear, and so the presentation
3. **Methodology section** I suggest to divide the method section into subsections such as: study design and setting, population of the study, sampling and sample size, data collection, and statistical analysis. This division is important for readers.
4. **Results section:**

This part is good, I have no comments

5. **Discussion:**  satisfied
6. **Conclusion:** satisfied
